# 4385 The Pain and Social Experiences Project: Understanding the role of interpersonal trauma in pain

**DOI:** 10.1017/cts.2020.345

**Published:** 2020-07-29

**Authors:** Jennifer Pierce, Afton L. Hassett, Rick E. Harris, Jenna Goesling, Chad M. Brummett

**Affiliations:** 1 University of Michigan School of Medicine

## Abstract

OBJECTIVES/GOALS: Traumatic interpersonal experiences are associated with higher rates of chronic pain, increased pain severity and poorer functioning. The objective of this ongoing project is to obtain prevalence rates for various forms of interpersonal trauma among individuals with chronic pain, and to explore the potential mediating effect of heightened sensory and social sensitivity on the experience of pain. METHODS/STUDY POPULATION: Patients at Michigan Medicine between the ages of 18 and 65 complete an online survey. Patients are being recruited through a tertiary-care, outpatient pain clinic, as well as through an online health research portal. We aim to recruit 700 participants; we currently have 59.6% of our goal (n = 417). Participants also have the option to be included in a registry from which we can recruit for future studies. Approximately 85% of our participants have agreed to be in the registry. RESULTS/ANTICIPATED RESULTS: Preliminary data show that, of the 263 (63.4%) participants for whom data on chronic pain is available, 167 (63.5%) report chronic or persistent pain over the previous 3 months. Of these, 54% reported some form of childhood abuse or neglect. Approximately 41% reported four or more adverse childhood experiences. Additionally, of the 122 participants (73%) who were in a current romantic relationship, 20% reported some form of physical violence victimization from their romantic partner. We anticipate that interpersonal trauma will be associated with poorer perceptions of social relationships, higher sensory sensitivity, and higher perceived stress. DISCUSSION/SIGNIFICANCE OF IMPACT: The PASE Project parent study will be used to better understand prevalence rates for various forms of interpersonal trauma in our chronic pain population. Future analyses and studies will explore alternative pathways linking interpersonal trauma to the experience of pain through sensory and social sensitivity, which will inform interventions aimed at reducing pain among patients with a history of trauma.

